# Active Learning in Psychiatry Education: Current Practices and Future Perspectives

**DOI:** 10.3389/fpsyt.2020.00211

**Published:** 2020-04-23

**Authors:** Stefano Sandrone, Jimmy V. Berthaud, Chad Carlson, Jacquelyne Cios, Neel Dixit, Amtul Farheen, Jessica Kraker, James W. M. Owens, Gustavo Patino, Harini Sarva, Daniel Weber, Logan D. Schneider

**Affiliations:** ^1^ A.B. Baker Section on Neurological Education, American Academy of Neurology, Minneapolis, MN, United States; ^2^ Department of Brain Sciences, Imperial College London, London, United Kingdom; ^3^ Department of Neurology, University of Michigan, Ann Arbor, MI, United States; ^4^ Department of Neurology, Medical College of Wisconsin, Milwaukee, WI, United States; ^5^ Department of Neurology, Ohio State University, Columbus, OH, United States; ^6^ Department of Neurology, Weill Cornell Medicine, New York, NY, United States; ^7^ Department of Neurology, Lebanon VA Medical Center, Lebanon, PA, United States; ^8^ Department of Neurology, Tulane University School of Medicine, New Orleans, LA, United States; ^9^ Department of Neurology, Division of Pediatric Neurology, University of Washington, Washington, Seattle, WA, United States; ^10^ Department of Biomedical Sciences, Division of Neuroscience, Oakland University William Beaumont School of Medicine, Auburn Hills, MI, United States; ^11^ Department of Neurology, St. Louis University, St. Louis, MO, United States; ^12^ Stanford/VA Alzheimer's Center, Palo Alto VA Health Care System, Livermore, CA, United States; ^13^ Sierra Pacific Mental Illness Research Education and Clinical Centers, VA Palo Alto Health Care System, Livermore, CA, United States

**Keywords:** active learning, flipped classroom, psychiatry education, curriculum design, clinical reasoning, flipping the curriculum, flipping the classroom

## Abstract

Over the past few decades, medical education has seen increased interest in the use of active learning formats to engage learners and promote knowledge application over knowledge acquisition. The field of psychiatry, in particular, has pioneered a host of novel active learning paradigms. These have contributed to our understanding of the role of andragogy along the continuum of medical education, from undergraduate to continuing medical education. In an effort to frame the successes and failures of various attempts at integrating active learning into healthcare curricula, a group of educators from the A. B. Baker Section on Neurological Education from the American Academy of Neurology reviewed the state of the field in its partner field of medical neuroscience. Herein we provide a narrative review of the literature, outlining the basis for implementing active learning, the novel formats that have been used, and the lessons learned from qualitative and quantitative analysis of the research that has been done to date. While preparation time seems to present the greatest obstacle to acceptance from learners and educators, there is generally positive reception to the new educational formats. Additionally, most assessments of trainee performance have suggested non-inferiority (if not superiority). However, occasional mixed findings point to a need for better assessments of the type of learning that these new formats engender: knowledge application rather than acquisition. Moreover, this field is relatively nascent and, in order to ascertain how best to integrate active learning into psychiatry education, a framework for quantitative outcome assessments is needed going forward.

## From the “Sage on the Stage” to the Flipped Classroom

Active learning is an emerging trend within higher education. It exemplifies a move away from the traditional teacher-centric approach of having an expert standing in front of the group and imparting knowledge (1, 2). Instead, new active learning (and teaching) strategies aim at moving beyond the lower-level cognitive process of knowledge acquisition and comprehension during class time and into application and analysis of the topics (3). Despite a restructuring of the activities and time balanced between home and classroom, active learning does not necessarily imply the complete abandonment of the lecture format, but, instead, it refers to a range of activities. These include: pre-class reading assignments, problem-based learning, team-based learning, simulator-based learning, use of worksheets or personal response systems, Q&A sessions or mini-cases built into the lecture, small group tutorials, problem-solving sessions, or use of the flipped classroom.

**Figure 1:**
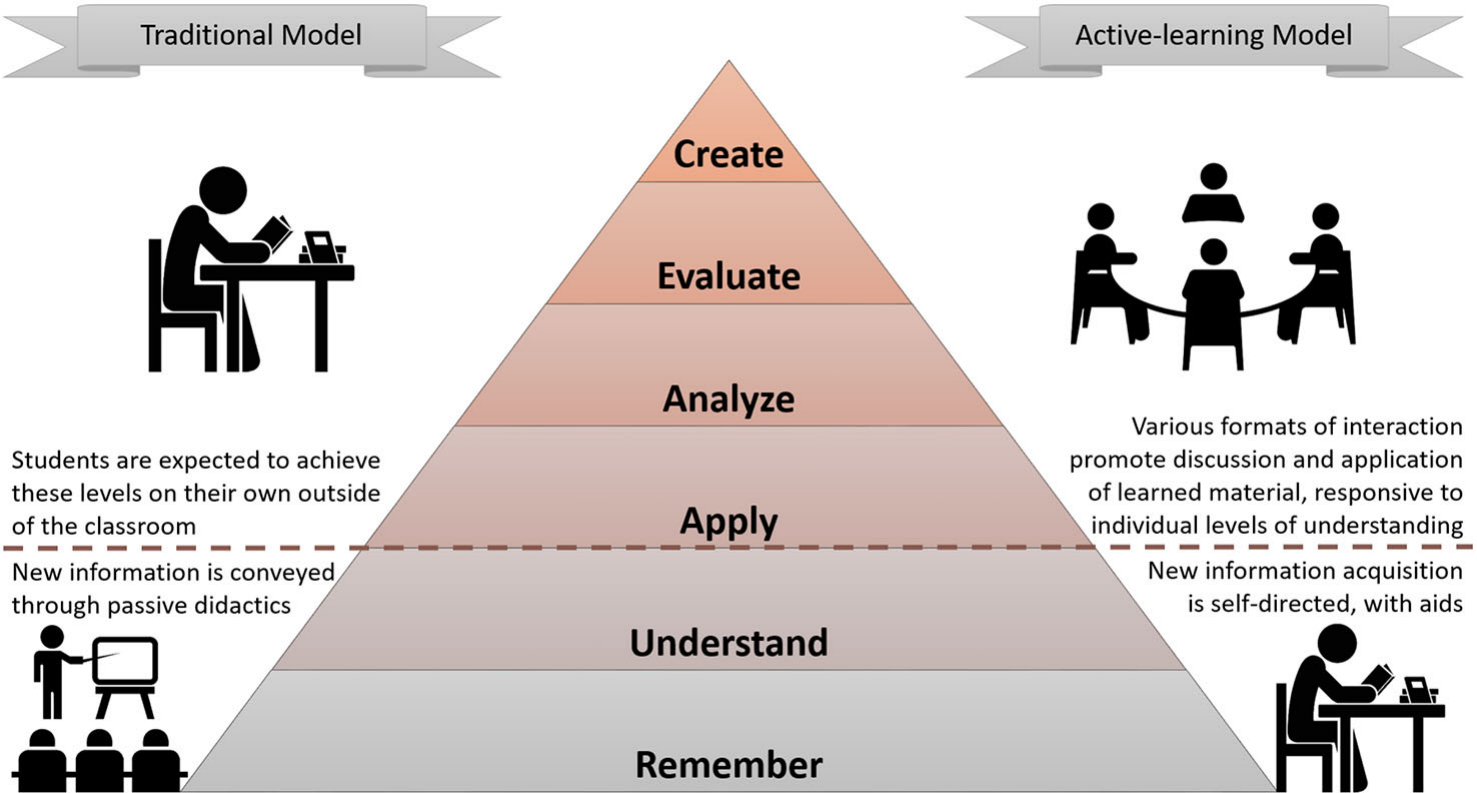
Bloom's taxonomy in a traditional versus a flipped classroom (5). In a traditional classroom, information is often provided *en masse* to learners in a passive format, with the expectation that higher-order learning is achieved through self-directed learning. Comparatively, the active learning in a flipped classroom promotes higher-order cognitive domain activities in a variety of novel formats (problem-based learning, etc.) that build upon learner-lead knowledge exposure outside of the classroom.

The flipped classroom, also labelled as reverse, inverse, or backwards classroom (4), is probably the most used among the active learning approaches (**Figure 1**). The learners are free to review the materials at their own pace and must be engaged in their learning process (6). While time at home is spent being initially exposed to the teaching material, face-to-face class time is dedicated to student-centered activities promoting active learning, under the supervision of a facilitator (2). Initially instituted in primary and undergraduate education, the flipped classroom has only recently made its way into the realm of medical education. Educators in various subspecialties are incorporating these methods into the curricula for students at all levels, from undergraduate to CME (7).

The putative success of active learning has encouraged the medical education community to explore what has already been learned through research on these methods. Among the subspecialties that have reported on their efforts, transitions of curricula to incorporate these modern methods in medical education have often resulted in hybrid approaches in which students are exposed to both flipped classrooms and traditional didactics (7, 8). However, an overarching reflection on what has been done so far in psychiatry is still lacking.

To this end, we provide a narrative review of active learning practices and highlight future perspectives related to their implementation and assessment along the andragogical spectrum of psychiatry education.

## Methods

Because of the relative lack of publications providing quantitative assessments of active learning models in medical education, and because of the inconsistent methods used to evaluate novel educational model performance, we sought to perform a narrative review, highlighting relevant quantitative data, where appropriate. To identify papers providing an evaluation of the application of active learning in psychiatry, a PubMed and Web of Science literature search was conducted in December 2018. The search used the terms “adult learning,” “flipped,” and “active learning” as well as “psychiatry,” “education,” “curriculum,” “course,” “medical,” “clinical,” “student,” “doctor,” “physician,” “resident,” “assessment,” “outcome,” and “evaluation,” combined into the following query: (adult learning OR flipped OR active learning) AND psychiatry AND (education OR curriculum OR course) AND (medical OR clinical) AND (student OR doctor OR physician OR resident) AND (assessment OR outcome OR evaluation). Of the results, in English, containing the query elements in either the abstract or the title, the initial review was limited to those publications indexed as articles, proceedings papers, and reviews. This strategy yielded 462 publications. Through review of the abstracts, we then excluded articles that did not evaluate the application of active learning strategies in psychiatry education. Following review of these publications, we applied a snowballing strategy in which we reviewed the cited references of the primary publications in order to identify any other relevant articles regarding active learning applied to health professional education, resulting in a total of 28 primary articles, included in this narrative review, along with more contemporary references to the relevant literature provided through the review process.

### Strategies for Incorporating Active Learning Models

Active learning offers the chance to develop students' higher-order cognitive skills and to engage them in processes that can improve health care delivery (4), provided it is sustainable (9). There is a reported appetite for the exploration of new educational models that involve interactive learning by integrating electronic/portable learning tools that meet modern learners in their realm of technological familiarity and on-demand access. For example, medical students who completed a psychiatry clerkship reported high use of electronic resources on an array of devices for learning and even indicated preference for such formats, compared to printed resources (10). Moreover, web-based instruction formats have the potential of tracking learning outcomes or even patient care on a longitudinal scale (11), and remote, interactive learning experiences, with expert faculty as moderators, can compensate for limited local expertise in certain topics (12). Regardless of the platform used, aligning the educational methods with learning objectives, within the broader context of clinical practice, is critical to the success of such active learning programs (13). Toward this end, a number of approaches have been attempted to realize an integrative, patient-centered approach (14).

### Team-Based and Problem-Based Learning

Team-based learning (TBL) enables active learning to be implemented within large classes (15). In a 5-week course, a TBL-based unit was given to 6–8 psychiatry residents across the training years of the psychiatry residency in the teaching of psychodynamic psychotherapy (16). Preparation with reading materials prior to class was followed by readiness assurance testing and the application of concepts, with faculty-led group discussion and case-based material. Residents rated the course on five items including: (1) clarity of objectives, (2) informativeness, (3) relevance to practice and to assessment, (4) degree of organization, and (5) overall value. All items were rated as excellent, with none scoring less than 4.84 (mean rating on a 5-point Likert scale, with 5 being the highest, and 1 being the lowest rating) (16). Faculty also appreciated the stimulating interaction with the residents they would not otherwise have had with the traditional lecture format (16). In a larger study, TBL significantly improved residents' rated classroom engagement and interactivity, although preparation was perceived as difficult by some learners (15).

In a different approach, students were involved in either a group discussion after assignment of reading (a problem-based learning approach) or passive instruction, followed by the making of a patient video as a team. While it was found that exam results were the same between the problem-based learning (PBL) and the lecture format group, medical students specifically cited increased readiness for the Objective Structured Clinical Examinations (OSCE) after the PBL exposure (17). This demonstrates that programs can incorporate PBL in a variety of ways. More generally, exploiting the similarity between a transdiagnostic psychiatric neuroscience approach and problem-based scenarios, which are common in clinical care, might further close the gap between current advances in psychiatric neuroscience and the education of trainees interested in the clinical translation (18, 19).

### Book Clubs, Social Media, and Other Unconventional Approaches

Although many andragogical tools fall within the active learning label, an important distinction has been made over the past two decades between deep and superficial learning (20). Deep learning has been described to entail more engaged learners, those with a genuine interest who seek more learning opportunities on their own and are self-driven to seek greater skills and knowledge (20). Inspiration of the engaged learner is arguably rooted in providing them with an outlet for discovery of subjects in which they have a genuine interest (i.e., giving learners the choice over subject matter in a journal or book club also represents a paradigmatic example of self-driven learning). The use of book clubs as an andragogical tool also falls within the active learning remit. Partially in response to the critical appraisal of psychiatry within the neuroscience paradigm, in which psychiatric disease is understood to be a result of purely biological processes, the rationale behind the adoption of this andragogical tool endeavored to teach psychiatry using a more constructionist philosophy, wherein the learners construct a concept of disease as a product of bio-psycho-social forces (21). Student responses were overwhelmingly positive: peer-peer learning relationships, exposure to multiple perspectives, and increased likelihood of reading the texts of their field were cited as strengths of this approach (21). This scheme was also used in combination with social media (i.e., Twitter) to generate a discussion about the book club prior to the meetings, although there is evidence that “passive” use of social media (e.g., Facebook) can have an adverse effect on affective wellbeing (22). Engagement with peers and the expert facilitator in online discussions further fanned interest through widened participation, thereby promoting a more inclusive approach beyond the classroom (21).

### Activating Learners as Educators

Another novel way in which the psychiatry literature has commented on the effort to involve active learning relates to the role of Education Chief Residents, where senior residents in psychiatry have defined roles in the education of students engaged in active learning (23). Duties of Education Chief Residents tend to include observing students interviewing a patient and then providing an opportunity for feedback and discussion, as well as providing tutoring for at-risk students, as identified by the treatment team, and recruiting other residents and faculty to intensively focus on improving the student's performance. In Ohio, where this was implemented to allow Education Chief Residents 10–12 hours per week fulfilling this role, the students rated the program highly in multiple domains: comfort going to the Education Chiefs with any difficulty (4.25), time allotted by the Education Chiefs for individuals seeking help (4.16), and feeling that the Educational Chiefs were a beneficial addition (4.32) (23). Additionally, the Education Chief Residents felt similarly gratified, based on reports of improvement in teaching and communication skills, administrative skills, and appreciation for the foundations of the Liaison Committee on Medical Education (LCME) requirements.

### The Case for Active Learning Models

From a more general perspective, a meta-analysis of 225 studies in undergraduate courses showed active learning as students' preferred teaching and learning method (24). Remarkably, active learning compared to traditional methods resulted in a 6% (0.47% SD) increase in exam scores, with students being 1.5 times as likely to fail exams using the traditional lecture-based format. In contrast, applications of active learning methods in psychiatry education are often more varied in both curricular format and outcome assessments, making comparisons somewhat difficult. Nonetheless, qualitative and quantitative measures reported to date suggest that active learning approaches are beneficial in psychiatry curricula.

### Qualitative Perspectives

A relatively recent literature review analyzed four different reviews and meta-analyses of active learning in higher education (25). Of the studies explored, three resident-level works and five reports at the undergraduate medical education level were reviewed (25). The residency-level studies generally found positive reviews of active learning approaches by the resident learners, although a smaller study of 5–8 residents who were given two, 6-month-long, problem-based learning (PBL) courses generated mixed reviews (26). In line with studies on active learning in other healthcare fields and specialties, negative comments centered around lack of available time to prepare for the studies on active learning sessions, residents' preference for traditional lectures, and suggestions to offer active learning to senior level residents (rather than those in their junior years of training) (7, 8, 25).

At the medical student level, active learning approaches have generally been limited to the length of a clerkship—around 1 month. To accommodate the educational needs of students in a time-limited clerkship, one group applied “blended learning”: a series of video lectures were assigned to the students, which had an associated graded online discussion board as a forum for questions and explanations, followed by face-to face sessions with case-based teaching (25). This resulted in high-quality student engagement as rated by the educators, who also reported their own unfamiliarity with active learning techniques as a weakness of the program (25). Also, the impact of active learning methods on attitudes toward psychiatry involved in an advanced Psychopharmacology elective was assessed. Student involvement was engendered by devoting half of each 2-h session to a student presentation and the other half to instructor-facilitated class discussion (27). Prior to each discussion, they were given a list of recommended readings and asked to read and critically evaluate 1–2 selections from the list. Students were then given the Attitudes Towards Psychiatry – 30 (ATP-30) questionnaire, and comparison was made between students’ responses before and after taking the course (27). Attitudes toward psychiatry improved significantly overall, with greatest improvement in domains assessing attitudes regarding the possibility of treatment and the identity of psychiatry as a biologically-based discipline (27).

### Quantitative Perspectives

An effort to improve the understanding of mental health concepts in baccalaureate nursing students introduced a number of active and self-directed learning methods—mental health scenario simulations aligned to classroom content and online, interactive case studies—into a curriculum (28). As a result, all the students completed the high-fidelity simulation checkoffs and achieved the clinical performance expectations, with 90% of them surpassing the minimum expectations for performance in mental health content (28). In the domain of resident education, one study sought to explore the effect of psychiatry resident-led review sessions in preparation for the Psychiatry Resident In-service Training Examination (PRITE exam) (29), a moderate predictor of the performance on the American Board of Psychiatry and Neurology (ABPN) Certification Examination in psychiatry. These 2-hour sessions included resident-prepared and delivered presentations, followed by a game show-style hour of questions and answers, in which teams engaged in a friendly competition that incorporated brief discussions of the rationale behind the correct responses. Overwhelmingly, the residents felt that both the prepared presentations—which utilized active learning for the residents who prepared the materials—and the game show-based components of the program were helpful (29). However, no significant difference was found on the overall PRITE scores when comparing performance to previous years in which lecture style review was given (29). But, on subsection analyses, there was a significant 9% decrease in the neurology subscores on the PRITE in the year of the flipped classroom review and a small, but non-significant, increase in psychiatry subscores (29). Given that neurology was not covered by the review program, the active learning approach may have actually offset a decline in overall scores in the year of the program, or it may not have made an appreciable difference in the PRITE scores on the covered psychiatry topics.

While there are multiple possible explanations for this observation, including heterogeneity in the residency representation from year to year, and the diversion of studying time from neurology to psychiatry topics, one possible explanation may be that flipped classroom models are superior at teaching to application- or problem-based mastery rather than the fact regurgitation that standardized tests often capture (29). Toward this end, some critics of National Board of Medical Examiners (NBME)-style exams cite that these examinations do not assess the deeper learning promoted by active learning methodologies (30). In addition, these types of exams do not measure interpersonal, communication, or professionalism skills. In line with this, other training programs have found a discordance between objective knowledge assessment scores on standardized testing when comparing active learning techniques and traditional educational methods. It may reflect a weakness of standardized tests developed for knowledge assessment (rather than application) as an outcome measure. Thus, identifying successful assessment methods of modern educational formats, as well as more effective measures of residents' satisfaction and the impact on their clinical competence, is fundamental (5, 31).

### Critical Discussion: Limitations, Challenges, and Opportunities

Here we sought to provide a narrative summary of the state of the field, with regard to active learning applications in psychiatry education. While the last two decades have seen a growth in the applications of validated strategies along the continuum of psychiatric education, active learning is not the panacea for all educational problems (6, 32). Challenges and opportunities for the application of new educational models incorporating heutagogical concepts to psychiatry education are similar to those faced by other medical specialties (7). Moreover, despite broader application of modern educational practices in psychiatry education, there is a paucity of publications by which to assess their efficacy. This inherently limited our study to a narrative review, focusing more on qualitative assessments rather than being able to apply meta-analytic insights from the handful of studies which reported non-comparable outcome metrics.

Nonetheless, a number of relevant insights can be derived from the existing body of literature that can help guide development and assessment of curricula integrating modern educational strategies into psychiatry education. Most learners seem to appreciate the new educational models, citing enthusiasm for the flexibility of content exposure, novel educational formats, and in-classroom engagement, but a number of limitations still exist. Most notably, the amount of time required to prepare for classroom sessions is a common complaint of learners, while lack of preparation for developing and implementing active learning curricula is a concern of educators. Reaching equilibrium between training requirements and personal interests might be a possibility to explore, especially given the focus of active learning on learner-driven educational change (33). It is interesting to note, however, that the front-end study time required to gain sufficient exposure to material is often not equivalent to the amount of time spent in traditional didactics. This ultimately may suggest that the expectation of higher-order cognitive tasks (as knowledge application) following passively delivered lectures may not actually be happening, given the discontent at the “new” workloads required in active learning curricula. Additionally, sharing information about new approaches—through best practices in both content development and delivery formats—across psychiatry programs is essential to widely “test” and, eventually, promote teaching innovations in psychiatry education (34). Moreover, the first step in defining outcomes that accurately measure curricular performance and allow for comparison is to recognize that modern educational approaches that focus on application of knowledge and more ineffable qualities (such as interpersonal communication, professionalism, and the like) may underperform when assessed through the lens of knowledge acquisition. In addition to this, as recently shown by Deslauriers and colleagues, students might not have the perception of “learning” with active learning approaches (35), therefore we need to share the rationale behind curricular changes with them.

A further hidden dimension, accounting for many of the aforementioned examples, deals with the “cost” of active learning as well as its implementation—factors ranging from staff training to technological support and the creation of new didactic materials—which might have an impact on the overall feasibility and sustainability of these teaching approaches (9), and can genuinely represent a concern among educators. However, access to the full range of modern educational resources may vary depending upon higher education/medical context. Toward this end, there is a risk of creating and/or widening existing educational barriers, particularly when considering resource limitations on a more global scale. Nevertheless, the ingenuity of educators who have addressed unique challenges to the implementation of active learning in various educational environments highlights the potential for overcoming obstacles to andragogical curricular development and deployment, even in the most challenging environments. In this vein, looking to extra-clinical educational applications of active learning can highlight novel approaches that overcome some of the traditional obstacles to such pedagogy. For example, some have addressed the limitations inherent to more foundational courses expected to cover large volumes of knowledge in large auditoriums of anonymized, disengaged learners by integrating mini-cases into jam-packed, fast-paced core courses (36). The wide variety of approaches offers the chance to selectively adopt what can best suit the needs of the learners, the curriculum, and the contextual resources, thus ensuring at least a low “activation energy” feasibility-wise across curricula, globally. Fortunately, these questions are being addressed, as evidenced by accumulating reports about active learning approaches in third world and developing countries, which “have not been left behind” (37, p147). Among these examples, although outside the field of psychiatry, are a flipped-classroom model “designed for developing universities in developing countries” for first-year students (38, p597) and a flipped-classroom model for high school students in developing countries (39).

Finally, as modern educational formats have shown promise not only in psychiatry, but also in other healthcare-related fields, a first step to discern their true value relies upon a more consistent method of assessment. Regardless of the aforementioned design elements used, any significant curricular modification should incorporate objective metrics into their study design to ensure external comparisons and meta-analytic assessment of curricular performance. Beyond evaluating stakeholder perceptions, a few areas in which the field should focus its efforts include explorations of what content is most amenable to these modern educational formats (e.g., pharmacotherapies vs psychotherapeutic strategies), as well as what audience is most appropriate for them (e.g., those with foundational knowledge vs those without). In considering these outcomes, we must also bear in mind the evolving educational regulatory field, where performance is no longer strictly measured by scores on a test, but encompasses a number of valuable traits (e.g., professionalism) and abilities (e.g., application rather than fund of knowledge) that are currently difficult to measure.

## Conclusions

In sum, the reception of modern educational formats in psychiatry has generally been positive, due to stakeholder satisfaction with curricular changes tending to outweigh the additional burdens imposed by these programs. However, the relative dearth of quantitative studies identified by this narrative review highlights a need for more rigorous evaluation of curricula to determine how to best apply active learning in psychiatry education.

## Author Contributions

SS and LS contributed to the conception and design of the study. SS, JB, CC, JC, ND, AF, JK, JO, GP, HS, DW, and LS contributed to the acquisition and analysis of data. SS, JB, CC, JC, ND, AF, JK, JO, GP, HS, DW, and LS contributed to drafting the text and preparing the figures.

## Disclaimer

The contents do not represent the views of US Department of Veteran Affairs or United States Government.

## Conflict of Interest

SS receives royalties from Oxford University Press (USA). JO receives honoraria for CME question writing from the journals *Neurology* and *Continuum*. HS has 5% support from the Michael J Fox Foundation, received clinical trial support from Biogen, Insightec, and Lundbeck Pharmaceuticals, and has received some internal funding from Cornell. She has also served on advisory boards for Merz and Amneal Pharmaceuticals and an independent video rater for Neurocrine Neurosciences. LS was supported by the Office of Academic Affiliations, Advanced Fellowship Program in Mental Illness Research and Treatment, Department of Veterans Affairs.

The remaining authors declare that the research was conducted in the absence of any commercial or financial relationships that could be construed as a potential conflict of interest.
